# Silicon Integrated Dual-Mode Interferometer with Differential Outputs

**DOI:** 10.3390/bios7030037

**Published:** 2017-09-14

**Authors:** Niklas Hoppe, Pascal Scheck, Rami Sweidan, Philipp Diersing, Lotte Rathgeber, Wolfgang Vogel, Benjamin Riegger, Alexander Southan, Manfred Berroth

**Affiliations:** 1 Institute of Electrical and Optical Communications Engineering (INT), University of Stuttgart, 70569 Stuttgart, Germany; pascal.scheck@gmail.com (P.S.); rami.sweidan@student.uni-tuebingen.de (R.S.); philipp.diersing@outlook.com (P.D.); lotte.rathgeber@int.uni-stuttgart.de (L.R.); w.vogel@int.uni-stuttgart.de (W.V.); berroth@int.uni-stuttgart.de (M.B.); 2 Institute of Interfacial Process Engineering and Plasma Technology (IGVP), University of Stuttgart, 70569 Stuttgart, Germany; benjamin.riegger@igvp.uni-stuttgart.de (B.R.); alexander.southan@igvp.uni-stuttgart.de (A.S.)

**Keywords:** integrated photonics, lab-on-a-chip, dual-mode, interferometer

## Abstract

The dual-mode interferometer (DMI) is an attractive alternative to Mach-Zehnder interferometers for sensor purposes, achieving sensitivities to refractive index changes close to state-of-the-art. Modern designs on silicon-on-insulator (SOI) platforms offer thermally stable and compact devices with insertion losses of less than 1 dB and high extinction ratios. Compact arrays of multiple DMIs in parallel are easy to fabricate due to the simple structure of the DMI. In this work, the principle of operation of an integrated DMI with differential outputs is presented which allows the unambiguous phase shift detection with a single wavelength measurement, rather than using a wavelength sweep and evaluating the optical output power spectrum. Fluctuating optical input power or varying attenuation due to different analyte concentrations can be compensated by observing the sum of the optical powers at the differential outputs. DMIs with two differential single-mode outputs are fabricated in a 250 nm SOI platform, and corresponding measurements are shown to explain the principle of operation in detail. A comparison of DMIs with the conventional Mach-Zehnder interferometer using the same technology concludes this work.

## 1. Introduction

The increasing demand for point of care diagnostics and highly sensitive monitoring in today’s process technology has led to huge efforts in miniaturizing optical sensors. Additionally, the attractive preliminary studies of integrated Raman sensors [1], chip-integrated interferometers that are sensitive to refractive index changes, are promising candidates for diverse applications. Surface functionalization on top of waveguides acts as the recognition element for specific substances [2]. By implementing photodiodes and read-out electronics on the same SOI chip, a compact sensing platform with a large number of interferometers in parallel can be built, which is a key element for a lab on a chip. Compared with the state-of-the-art sensitivity of such non-resonant interferometers [3], the so-called dual-mode or bimodal interferometer (DMI) represents a fairly unknown but very promising design. The DMI uses two optical paths in a single waveguide by utilizing two different optical modes. These two modes of a single waveguide are confined differently, resulting in different penetration depths of the evanescent fields in the cladding or environment. Because it requires only one waveguide, this design is much more compact than its counterpart, the Mach-Zehnder interferometer (MZI). In addition, further isolation layers to passivate the reference waveguide are avoided. Moreover, both modes in a DMI are at nearly the same temperature, due to their close proximity; they are not thermally separated as in common MZI designs. In this work, operating principles for an extended DMI design are presented, which allow the determination of the phase relation of the two modes with a single wavelength measurement [4]. The paper is structured as follows: A short summary of DMI designs is given to illustrate the advantages and drawbacks of existing designs. The theory of basic and single wavelength operation is explained in detail in Section 3. The performance parameters of the DMI design, such as insertion loss (IL) and extinction ratio (ER), are analyzed in Section 4. A comparison to MZIs using the same technology (Section 5) and a summary concludes this work. 

## 2. Designs of Dual-Mode Interferometers

Various DMI layouts can be found in scientific publications. While this overview is focused on DMI designs utilizing only the fundamental and second-order quasi-transverse electric (TE) modes, there are further designs which use either higher-order modes [5], or two different polarizations in the dual-mode section [6]. The basic DMI types utilizing the two lowest-order TE modes are depicted in Table 1.

The main difference between the designs is the conversion mechanism from a single mode at the input to the desired two modes in the two-mode section of the interferometer, and vice versa. In the first DMI type, the mode splitter and combiner are realized as vertical waveguide steps. Type 2 uses a vertical step at the input and a Young interferometer configuration, including a free space section, at the output. An external CCD array detects the interference pattern. In type 3, the mode splitter and combiner are realized by asymmetric lateral waveguide steps. No additional etching process is required here. In the design of type 4, a special long-period grating is used to convert the modes, enabling a theoretical excess loss of mode conversion of less than 0.22 dB. The optical coupling to the single mode waveguides at input and output is accomplished through further gratings. For type 1, 2 and 4 DMIs, the depth of the vertical step in the waveguide or of the grating grooves, respectively, is a critical parameter, and has to be controlled carefully during the fabrication process.

**Figure 1:**
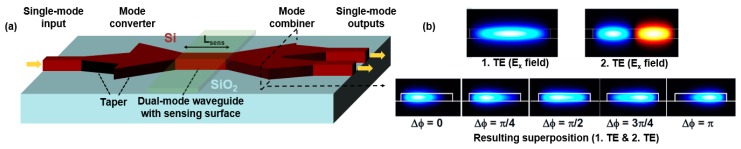
Buildup of the DMI with two differential DMI outputs (type 7). The schematic view (**a**) and the field profiles (**b**) of the combiner section are shown.

The design of type 5 uses laterally displaced fibers on common grating couplers. This allows the excitation of two modes with arbitrary power ratio, defined by the displacement of the fiber in respect to the waveguide axis. This setup is very flexible in terms of mode excitation; however, it requires proper and stable alignment of the fibers. For large arrays of interferometers in labs on a chip, the Young configuration of type 2 and the configuration with displaced fibers (type 5) are less convenient, due to the additional space and adjustment requirements. In type 6 and 7, the mode splitters and combiners are separate parts that have fixed dimensions. Tapers are used to adapt to different waveguide widths. This makes the design of the dual-mode section independent from other geometries, such as input waveguide width, resulting in a modular DMI design. Moreover, the tolerance of the splitter and combiner to imperfections of the fabrication process is enhanced compared to the type 3 DMI due to the enlarged splitter and combiner dimensions, and no critical vertical etching step is required. The type 7 DMI is an extended version of type 6, with a mode combiner with two outputs. A more detailed view is depicted in Figure 1a. The individual output powers of the two outputs are inverse to each other, due to the resulting superposition of the two phase-shifted modes (see Figure 1b). The sum of the two powers is always constant, and equal to the input power, assuming an ideal transmission with no losses.

The type 7 DMI has a slightly higher excess loss of the mode conversion in the combiner compared to types 4 and 6, but with the two differential outputs it is possible to determine the phase condition of the interference in a range of π [4] with only one measurement at a single wavelength, rather than evaluating the transmission spectrum in a broad range. Thus, no tunable laser sources are required to operate the DMI in a sensor system.

## 3. Operating Principle of the Dual-Mode Interferometer with Differential Outputs

For describing the resulting sensitivity of a type 7 DMI, a distinction can be made between surface sensitivity and homogenous bulk sensitivity as in [5]. The latter can be related to homogenous cladding changes, and is used in this work to explain the operating principle. The homogenous bulk sensitivity *S_bulk_* is defined as the change of the phase difference *Δ*φ over the change of the refractive index of the cladding, and can be expressed by

(1) $$S_{bulk}=\left|\frac{\partial \left(\Delta \phi \right)}{\partial n_{c}}\right|=\frac{2\pi L_{sens}}{\lambda_{0}}\left|\frac{\partial \left(n_{m1}-n_{m2}\right)}{\partial n_{c}}\right|=\frac{2\pi L_{sens}}{\lambda_{0}}\eta_{bulk},$$

where $\partial n_{c}$ is the change of the refractive index in the sensing region with the length *L_sens_*. The effective refractive indices of the fundamental and second-order modes are *n_m1_* and *n_m2_*, respectively. The phase difference between the two modes changes with the refractive index of the cladding due to different modal sensitivities resulting from their modal profiles. This fundamental behavior is also the same for other DMIs [5], and the sensitivity of a waveguide cross-section can be characterized by the intrinsic bulk sensitivity to changes in the cladding layer η*_bulk_*, which can be larger than 50%. However, depending on the requested *Δ*φ, the optical signals in the dual-mode waveguide of a type 7 DMI can be coupled to output 1 or output 2 (see Figure 1b).

**Figure 2:**
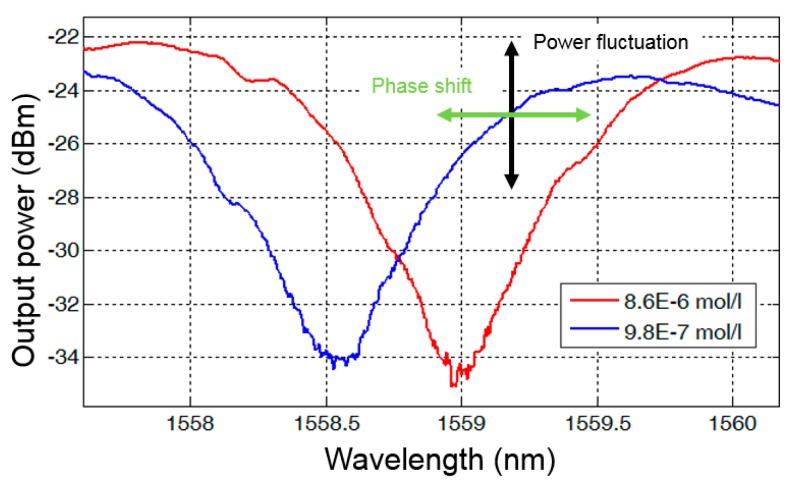
Measurements of a type 5 DMI with two different concentrations of Pb(II) ions in water. The optical input power is 0 dBm.

When only one output port (as in DMIs of type 1 or types 3–6) is available, the determination of phase difference *Δ*φ must be done by evaluating the transmission spectrum in a certain wavelength range. This is illustrated in Figure 2 with an exemplary detection of Pb(II) ions from Pb(NO_3_)_2_ in water (see Appendix A). The transmission minimum moves towards a longer wavelength due to the phase shift induced by the increased concentration of Pb(II) ions. At the same time, the spectrum is shifted upwards to higher transmitted power levels, i.e., the total losses also depend on the concentration of Pb(II) ions. With a measurement of the transmitted power at only a single wavelength there is neither the possibility to distinguish between phase shift induced change in transmission and loss dependent change in transmission nor the possibility of perceiving input power fluctuations.

**Figure 3:**
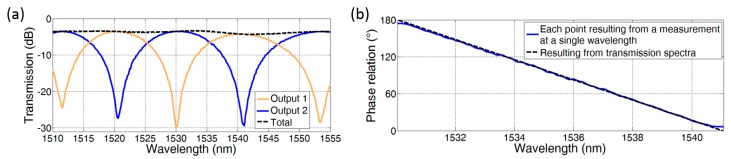
(**a**) Transmission spectra of two differential DMI outputs (type 7) and the corresponding sum. The dual-mode waveguide length and width are 200 μm and 625 nm, respectively; (**b**) Resulting phase relation.

The evaluation of the two differential outputs of a type 7 DMI results in a measured transmission spectrum as shown in Figure 3a. The antiphase transmission characteristic is clearly visible, and the sum of the two output powers (dotted line) is constant.

Assuming an ideal modulation transfer curve with ER = ∞, which is frequently used for MZIs, the phase difference between the two modes is calculated from the two output powers *P*_1_ and *P*_2_ by

(2) $$\Delta \phi \approx 2\cos^{-1}\sqrt{\frac{P_{1}}{P_{1}+P_{2}}}.$$

The result calculated from the shown formula is depicted in Figure 3b. The calculation is done over an interval from minimum *P*_1_ at 1530 nm to maximum *P*_1_ at 1541 nm, where the phase relation *∆*φ ranges from 180° to 0°. The well-known problem of estimating a phase shift larger than π can be circumvented [13] by either using a transmission measurement versus time or else by placing several interferometers with different sensing lengths in parallel, which can be done very easily with DMIs. 

## 4. Performance of Type 7 Dual-Mode Interferometers

**Figure 4:**
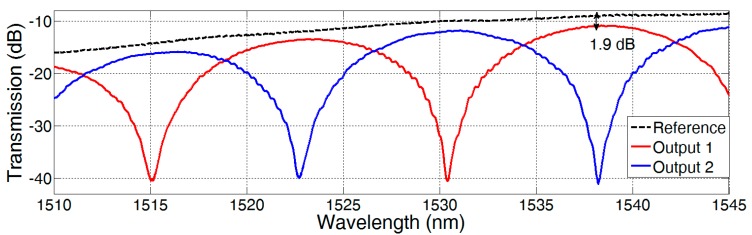
Transmission spectra of two differential DMI outputs (type 7) with straight output tapers and the transmission spectrum of the reference. The dual-mode waveguide length and width are 200 μm and 625 nm, respectively. The transmission of a reference structure is shown, which is used for the determination of the IL.

Apart from the sensitivity of a DMI, there are several important operational parameters, such as IL and ER. To determine these parameters, type 7 DMIs were fabricated in the 250 nm SOI platform at IMS CHIPS Stuttgart. The transmission spectra of the two outputs of a DMI with a 200 µm long dual-mode waveguide are depicted in Figure 4. 

The resulting IL of 1.9 dB at a wavelength of 1538 nm consists of two times the excess loss of the mode converters and the waveguide and taper losses. Assuming small waveguide and taper losses, the measurement is in good accordance to the simulation of the excess loss of mode conversion in [12], which results in 0.9 dB at 1550 nm. 

**Figure 5:**
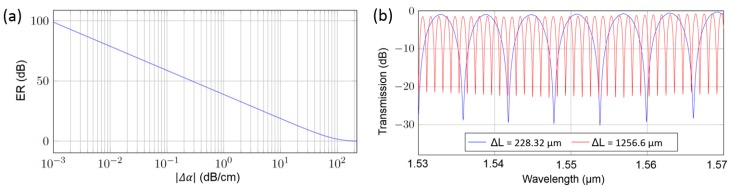
(**a**) Extinction ratio versus signal loss difference *Δ*α for a 1 mm long DMI; (**b**) Transmission spectra of two exemplary MZIs with corresponding arm length differences. The decreased extinction ratio can be used for the calculation of the waveguide loss.

In this measurement, the ER of the DMI is limited mainly due to the dynamic range of the power meter (Agilent 81625A). In general, the ER of the DMI sensor depends on the achievable ratio of mode powers *P*_1_/*P*_2_ at the combiner [12]. Due to different mode profiles in the sensing region, usually the mode losses differ, which consequently decreases the ER (see Figure 5a). This effect is clearly visible in the measured transmission spectra of two MZIs with different arm length differences (see Figure 5b). The additional waveguide loss in one MZI arm decreases the ER.

Therefore, deeper insight in the mode loss mechanism and the estimation of the order of magnitude of especially the higher order mode losses helps in designing an attractive DMI with high ER and low IL. Several works treat the fundamental mode losses in Si waveguides, but there is less work on the experimental determination of the higher-order mode loss, which is also important for other systems, such as multimodal on-chip data links [14]. 

The loss $\alpha_{m2}$ of the second-order mode in a DMI is composed of common scattering and absorption losses $\alpha_{norm}$, and the power fraction leaking to the fundamental mode, expressed by $\alpha_{m2\to m1}$. To estimate the higher-order mode loss there are several options. The pure excitation of the interested higher-order mode in combination with the commonly used cut-back method is an easy-to-understand way. For this purpose, a DMI with two single-mode inputs can be used, acting as a kind of 2 × 2 multimode interferometer (MMI). Like in a tunable power splitter, the phase condition of the input signals controls the power ratio. With a suitable phase relation at the input, almost pure excitation of the higher order mode can be achieved. 

In this work, we use DMIs with different strip waveguide lengths L and DMI mode splitters, which are also used as mode combiners, with a theoretical input power ratio of 1. If $\alpha_{m2\to m1}$ is small enough, the measured ER can be used to calculate the difference between the higher-order mode loss and the fundamental mode loss *Δ*α by

(3) $$\left|\Delta \alpha \right|=\left|\alpha_{m2}-\alpha_{m1}\right|=\frac{20\log_{10}\left(\frac{\sqrt{ER}+1}{\sqrt{ER}-1}\right)}{L}\mathrm{dB}.$$

**Figure 6:**
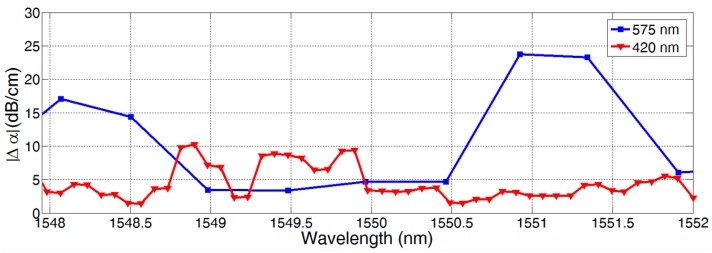
Loss difference versus wavelength for two fabricated DMIs with a waveguide width of 420 nm and 575 nm.

This calculation is done for DMIs with different dual-mode waveguide widths W, and ERs were extracted using the minima and maxima of the optical transmission spectrum close to 1550 nm. The resulting loss differences are depicted over wavelength in Figure 6.

Note that the waveguide thickness is always 250 nm and the waveguides are cladded by a 1 µm thick SiO_2_ layer. Utilizing measurements in the same technology resulting from the cut-back method for a width W of 400 nm and for the fundamental TE mode results in $\alpha_{m1}$ is equal to 3.3 dB/cm at 1550 nm. As a consequence, $\alpha_{m2}$ for W = 420 nm can be estimated to be smaller than 14 dB/cm in the analyzed wavelength range. Taking the smallest loss difference into account and assuming $\alpha_{m2}>\;\alpha_{m1}$ results in $\alpha_{m2}$ ≈ 4.5 dB/cm. The variation of the additional loss over wavelength may be closely associated with the influence of stitching errors caused by the e-beam lithography fabrication step. In addition, the wavelength dependency of the mode-to-mode coupling strength should be investigated in further works. In addition to the IL and ER, the thermal dependency is an important characteristic for sensor applications. For type 7 DMIs, the thermal dependency is mainly caused by the phase relation of both modes in the dual-mode waveguide, and can be minimized with a proper waveguide geometry as discussed in [12]. 

## 5. Comparison between DMI and MZI

**Figure 7:**
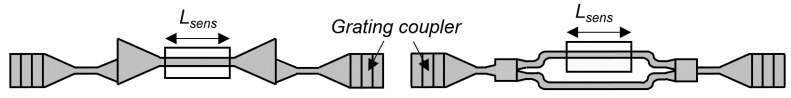
Schematic view of fabricated devices for the comparison of the DMIs with the MZIs.

To compare the performance of the DMI with the performance of MZIs, both interferometer types were fabricated using a 250 nm SOI technology with partially uncovered Si waveguide arms. The devices are shown in Figure 7. 

The in-coupling and out-coupling of the optical signals to single-mode fibers (SMF-28) is done by using identical aperiodic grating couplers. With the help of two different sensor region lengths *L_sens_* for each interferometer type, a determination of the additional loss caused by only the sensing region becomes possible. An increased sensor region length leads to a larger sensitivity of the device, although usually there is a compromise between loss and sensitivity of a designed interferometer. The decrease in loss given in dB and gain in sensitivity increase linearly with the sensor region length. Therefore, the product of the sensitivity and the reciprocal additional loss is calculated to make the comparison as fair as possible, and length-independent. The resulting figure of merit (FOM) is the bulk sensitivity per loss, which can be expressed by

(4) $$S_{bulk}/\alpha =\frac{2\pi \Delta L_{sens}\eta_{bulk}}{\lambda_{0}\;\Delta IL}$$

where *ΔL_sens_* is the difference of the sensor region waveguide length and *ΔIL* is the difference in IL of the two interferometers. This parameter and the other results are listed in Table 2 for DMIs and MZIs. 

Even if the IL for short MZIs is better than for short DMIs, this situation is reversed for longer devices, due to the different additional sensor region losses. This fact is reflected by the much higher sensitivity per loss FOM of the DMI. Following the calculated values in Table 2, the fabricated long DMIs are predominant, but the MZIs achieve a higher ER. Higher ER and lower losses are also achieved by the DMI with 420 nm waveguide width, but the influence of temperature on the phase difference is increased. Please note that these ILs depend strongly on the technology used, and this comparison is not valid for all technologies. 

## 6. Discussion and Conclusions

In this work, the operating principles of silicon integrated dual-mode interferometers for a stable single-wavelength operation are presented. The influence of an undetermined or fluctuating optical input power, as well as varying absorption in the sensor region, can be compensated by exploiting power measurements at a single-wavelength using power sum and ratio of two differential outputs. Using the latest designs, detection with high extinction ratio and low insertion loss is possible. The phase condition ambiguity can be circumvented by two or more interferometers with different sensor region lengths in parallel. In this work, the operating principle is discussed in detail, and the detection of Pb(II) ions in water is shown, which demonstrates a sensor application of the DMI. Further, procedures are presented to estimate the losses of higher-order modes in optical waveguides. In the technology utilized here the bulk sensitivity per loss given in dB^−1^ in the sensor region of dual-mode interferometers is more than twice as high as in MZIs. This makes the DMI attractive especially when very long sensing waveguides are required.

## Figures and Tables

**Table 1:** A comparison of different TE dual-mode interferometer designs is shown: The schematic buildups of the designs are depicted on the left. Corresponding assets and drawbacks of the design are shown on the right. Notes: 0 indicates large additional space requirements; + indicates minor additional space requirements; ++ indicates large on-chip arrays possible; * indicates theoretical values for balanced mode excitation.

		Reference/Year	DMI Array	Excess Loss of Mode Conversion	Single Wavelength Operation
**Type 1** (side view)	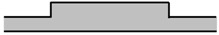	[7]/2009	**++**	0.5 dB *	**✕**
**Type 2** (side view)	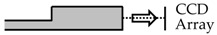	[8]/2011	**+**	unknown	**✓**
**Type 3** (top view)	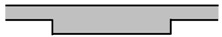	[9]/2014	**++**	0.5 dB *	**✕**
**Type 4** (side view)	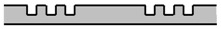	[10]/2014	**++**	< 0.22 dB *	**✕**
**Type 5** (top view)	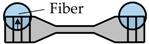	[11]/2015	0	< 4.2 dB	**✕**
**Type 6** (top view)	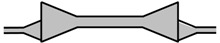	[12]/2016	**++**	0.25 dB * < 0.5 dB	**✕**
**Type 7** (top view)	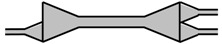	[4]/2017 this work	**++**	0.55 dB * < 2 dB	**✓**

**Table 2:** Resulting performance values for DMIs and MZIs close to 1550 nm. The intrinsic bulk sensitivity is simulated with the FIMMWAVE finite difference waveguide mode solver for a refractive index change of 0.01 and for the DMI as difference of the two intrinsic mode sensitivities.

Device	Waveguide Width	Sensor Region Length	Measured IL	Intrinsic Bulk Sensitivity	Measured ER	Bulk Sensitivity per Loss
MZI	250 nm	500 µm	1 dB	79%	>30 dB	1441 dB^−1^
		5000 µm	11 dB		>25 dB	
DMI	575 nm	500 µm	2.5 dB	44%	≈20 dB	3237 dB^−1^
		6400 µm	5.2 dB		≈10 dB	

**Figure A1:**
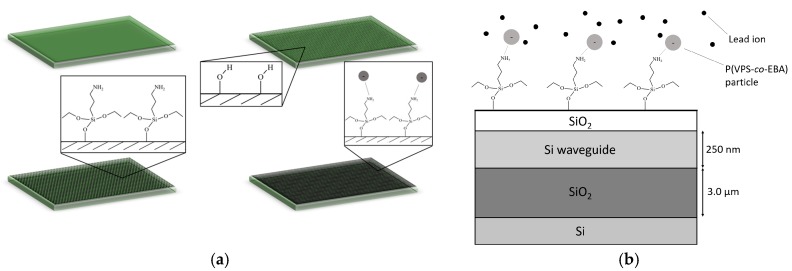
Fabrication of a P(VPS-co-EBA) particle covered dual-mode waveguide. (**a**) Fabrication steps; (**b**) Resulting layer stack.

**Figure A2:**
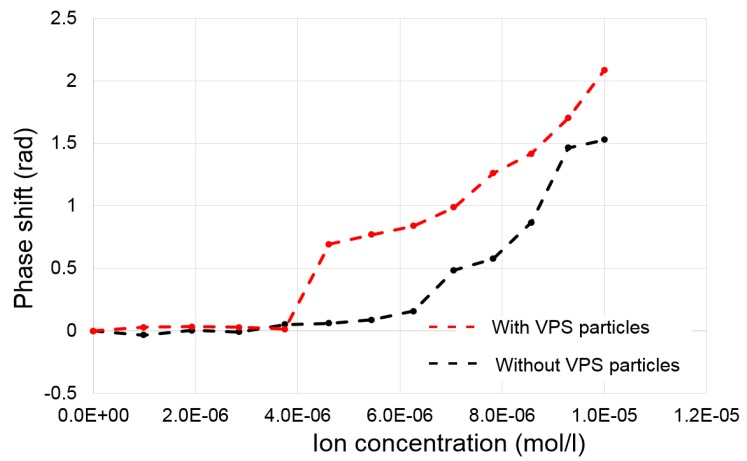
Measurement of the resulting phase condition with fixed fibers resulting from a particle-covered DMI and a not-covered DMI by varying concentration of Pb(II) ions in water. The evaluation of the phase condition is done using the minima in the transmission spectra close to 1540 nm. The temperature influence is reduced by recording the solution temperature and using a temperature coefficient of 0.1249 rad/K for the resulting corrected phase shift. The temperature coefficient can be decreased with an optimized waveguide geometry, as shown in [12].

## Appendix A

P(VPS-co-EBA) nanoparticles for coating of the sensor surface were prepared by miniemulsion polymerization using the monomer vinylphosphonic acid (VPS) and the cross-linker *N*,*N*′-ethylenebisacrylamide (EBA), according to Niedergall et al. [15]. For the coating of the sensor surface, the particles were redispersed in water, where the phosphonic acid groups present in the particles were partly deprotonated to negatively charged phosphonates. The Si waveguides were fabricated using 250 nm SOI technology at IMS CHIPS Stuttgart using e-beam lithography. Figure A1 illustrates the fabrication process for the particle layer. First, the chip of interest was cleaned with acetone, isopropanol and distilled water to remove organic impurities on the surface (Figure A1a, top left). Next, the chip surface was activated to increase the concentration of hydroxyl groups. This was accomplished by exposing the chip to a mixture of hydrogen peroxide solution (30%) and ammonium hydroxide solution (25%) in a volume ratio of 2:3 (*v/v*) at 70 °C for 40 min (Figure A1a, top right). After that, the chip was submerged overnight in a solution of (3-aminopropyl)triethoxysilane in ethanol, followed by a washing step in ethanol and drying (Figure A1a, bottom left). Thus, in contact with water, positively charged ammonium groups were present on the surface. Finally, the chip was immersed in the suspension containing the P(VPS-co-EBA) nanoparticles, and the particles adhered to the surface, probably due to electrostatic interactions of the oppositely charged ammonium and phosphonate groups. Thus, the sensor surface was coated with the particles via dip coating (Figure A1a, bottom right), and was then ready for measurements (Figure A1b).

The used particles already showed an enhanced Pb(II) ion adsorption property in other works [15], which can be ascribed to the generally strong interaction of Pb(II) ions with phosphonate and phosphate groups as documented by the very low solubility of lead(II) phosphate in water. In our studies, the sensor response and resulting phase shift, which is depicted in Figure A2, is enhanced in a specific working range by the particle layer compared to the same DMI without functional cladding.

Despite this enhancement, the repetition of the measurement with the same particles is challenging, and its purification will be the subject of further research.
